# Dynamic Distribution of Nuclear Coactivator 4 during Mitosis: Association with Mitotic Apparatus and Midbodies

**DOI:** 10.1371/journal.pone.0022257

**Published:** 2011-07-26

**Authors:** Alexandra Kollara, Maurice J. Ringuette, Theodore J. Brown

**Affiliations:** 1 Department of Obstetrics and Gynecology, Samuel Lunenfeld Research Institute, Mount Sinai Hospital, Toronto, Ontario, Canada; 2 Department of Cell and Systems Biology, University of Toronto, Toronto, Ontario, Canada; Virginia Tech, United States of America

## Abstract

The cytoplasmic localization of Nuclear Receptor Coactivator 4 (NcoA4), also referred to as androgen receptor associated protein 70 (ARA70), indicates it may possess activities in addition to its role within the nucleus as a transcriptional enhancer. Towards identifying novel functions of NcoA4, we performed an *in silico* analysis of its amino acid sequence to identify potential functional domains and related proteins, and examined its subcellular distribution throughout the cell cycle. NcoA4 has no known or predicted functional or structural domains with the exception of an LxxLL and FxxLF nuclear receptor interaction motif and an N-terminal putative coiled-coil domain. Phylogenetic analysis indicated that NcoA4 has no paralogs and that a region referred to as ARA70-I family domain, located within the N-terminus and overlapping with the coiled-coil domain, is evolutionarily conserved in metazoans ranging from cnidarians to mammals. An adjacent conserved region, designated ARA70-II family domain, with no significant sequence similarity to the ARA70-I domain, is restricted to vertebrates. We demonstrate NcoA4 co-localizes with microtubules and microtubule organizing centers during prophase. Strong NcoA4 accumulation at the centrosomes was detected during interphase and telophase, with decreased levels at metaphase and anaphase. NcoA4 co-localized with tubulin and acetylated tubulin to the mitotic spindles during metaphase and anaphase, and to midbodies during telophase. Consistent with these observations, we demonstrated an interaction between NcoA4 and α-tubulin. Co-localization was not observed with microfilaments. These findings indicate a dynamic distribution of NcoA4 with components of the mitotic apparatus that is consistent with a potential non-transcriptional regulatory function(s) during cell division, which may be evolutionarily conserved.

## Introduction

Nuclear receptor coactivator 4 (NcoA4), also known as androgen receptor associated protein 70 (ARA70), is a ubiquitously expressed protein shown to enhance the transcriptional activity of multiple ligand-activated nuclear receptors [1]-[7]. Similar to the prototypical p160 receptor coactivators, which include steroid receptor coactivator (SRC) 1, 2, and 3, NcoA4 contains signature nuclear receptor interaction motifs: an LxxLL motif located in the N-terminal domain and an FxxLF motif located in the central region of the protein [3], [6], [8]–[11]. However, unlike the p160 coactivators, NcoA4 lacks a recognized nuclear localization signal and localizes predominantly in the cytoplasm even following receptor ligand stimulation [4], [5], [8]. This cytoplasmic localization suggests that NcoA4 may participate in cytoplasmic events that enhance receptor activity/function [8], and that it has additional, as yet undefined functions independent of receptor activity.

NcoA4 lacks known structural or functional domains that would facilitate the prediction of additional functions with the exception of an N-terminus putative coiled-coil protein-protein interaction domain, as predicted by COILS (ExPASy proteomics: http://ca.expasy.org/). In this study, we performed a phylogenetic analysis of NcoA4 and examined its subcellular distribution as an initial step to identify novel functions of this unique protein. We provide evidence that NcoA4 is an evolutionarily conserved protein with no paralogs. NcoA4 localized to microtubules, undergoing dynamic changes in its distribution during cell-cycle progression, consistent with a potential action in chromosome segregation and/or cytokinesis.

## Materials and Methods

### In silico identification of NcoA4 genes and phylogenetic analysis

BLASTp searches of the Genbank database (http://blast.ncbi.nlm.nih.gov/Blast.cgi) has identified orthologs of *Homo sapiens* NcoA4 (human; GenBank accession no. CAI14478.1) ranging from cnidarians to mammals: *Mus musculus* (mouse; GenBank accession no. EDL24830.1), *Gallus gallus* (chicken; GenBank accession no. NP_001006495.1), *Xenopus laevis* (African clawed frog; GenBank accession no. NP_001089238.1), *Danio rerio* (zebrafish; GenBank accession no. NP_957423.1), *Strongylocentrotus purpuratus* (sea urchin; GenBank accession no. XP_790295.2), *Saccoglossus kowalevskii* (acorn worm; GenBank accession no. XP_002741848.1), *Nematostella vectensis* (starlet sea anemone; GenBank accession no. XP_001638385.1). Gene identity was based on E-values ranging from <e^−168^ to 2e^−10^, as E-values ≤1e^−10^ are considered an experimental confirmation of gene identity. A rooted phylogenetic tree was generated by Clustal W and Phylip's Drawtree software using full-length NcoA4 orthologs [12], [13].

### Cell culture and transfections

Green African monkey kidney cells COS, human breast T47D and ovarian ES2 cancer cells (ATCC, Manassas, VA, USA) were grown in Dulbecco's modified Eagle's medium supplemented with 10% heat-inactivated fetal bovine serum, 100 units/ml penicillin, 100 g/ml streptomycin and 0.625 µg amphotericin B (Gibco-BRL, Gaithersberg, MD, USA). The medium for T47D cells was supplemented with 0.16 U/ml insulin. Methyl-(5-(2-thienylcarbonyl)-1H-benzimidazol-2-yl) carbamate (nocodazole) was obtained from Sigma Chemical Co. (St.Louis, MO, USA).

An expression construct for full-length NcoA4 cDNA (pCRII-NcoA4) was generated as previously described [4]. pECFP-NcoA4 was generated by ligating full-length NcoA4 into pECFP-N1 vector (Clontech, Mountain View, CA, USA) at the *EcoRI/BamHI* restriction sites and was sequence verified. T47D and COS cells were transiently transfected with pECFP-N1 or pECFP-NcoA4 cDNA using Lipofectamine™ 2000 (Invitrogen, Burlington, ON, Canada) according to the manufacturer's recommended protocol.

### Co-immunoprecipitation and Western blot analysis

For co-immunoprecipitation assays, T47D and COS cell lysates were harvested as previously described [4] and were incubated overnight at 4°C with 1 µg/ml NcoA4 rabbit polyclonal antibody (H300; Santa Cruz Biotechnology, Santa Cruz, CA, USA), *α*-tubulin mouse monoclonal antibody (Sigma), or Living Colors (anti-CFP) rabbit polyclonal antibody (Clontech). Co-immunoprecipitation and Western blot analysis were performed as previously described [4]. T47D and COS cell lysates were used as a positive control. The membranes were probed with anti-NcoA4 (1∶200), or anti-*α*–tubulin (1∶200) antibody as indicated for each experiment.

### Immunofluorescence staining and imaging

T47D, COS and ES2 cells were grown in 8-well chamber slides. The cells were washed with PHEM buffer (160 mM PIPES, 50 mM HEPES, 10 mM EGTA, 2 mM MgCl_2_, pH 6.9) and incubated with 0.5% Surfact-Amps® X-100 (Pierce, Rockford, IL, USA) in PHEM buffer for 90 seconds. Cells were then fixed in PHEM buffer containing 3.7% formalin, 0.25% glutaraldehyde, and 0.5% Surfact-Amps® X-100 for 15 min. The slides were washed in PBS and incubated with 0.1% Surfact-Amps® X-100 in PBS for 15 min followed by blockage in 10% normal goat serum (Vector laboratories, Burlingame, CA, USA) for 1 h. The slides were incubated with rabbit (H300,1∶100), mouse (1∶100; Novus Biologicals) or goat D19 (1∶100) or Q19 (1∶2000) (both from Santa Cruz Biotechnology) anti-NcoA4, mouse anti-α-tubulin (1∶200), anti-β-tubulin (1∶200: Sigma), anti-acetylated tubulin (1∶200; Sigma), anti-γ-tubulin (1∶200; Sigma), anti-actin (1∶200; Sigma), or anti-Plk1 (1∶200; Santa Cruz Biotechnology) antibodies overnight at 4°C. Slides were then washed in PBS and incubated with Alexa Fluor® 488 donkey anti-mouse IgG or Alexa Fluor® 594 donkey anti-rabbit IgG (both 1∶200; Molecular Probes, Eugene, OR, USA) for 1 h. To visualize chromatin, sections were incubated for 2 min with 4′-6-diamidino-2-phenylindole (DAPI) (2 µg/ml). The slides were rinsed in PBS and coverslips were mounted using 1,4-diazabicyclo[2.2.2]octane (DABCO; Sigma). Preabsorption studies were performed with Q19 NcoA4 antibody preincubated with a 5-fold molar excess of the immunizing peptide (Santa Cruz Biotechnology).

Images shown in Figures 2 (panels D to F and J to L), 5 (panels D to F) and 8 (panels D to F) were generated by deconvolution microscopy using a CoolSnap HQ CCD camera with Olympus lens N.A. 1.35 (40X to 100X) and iterative process for deconvolution algorithm as designated by Applied Precision Inc (Washington, DC, USA). The images shown in Figures 2 (panels A to C and G to I), 4, 5 (panels A to C and G to L), 6, 7, 8 (panels A to C) and 9 were generated by confocal microscopy using a Hamamatsu C9100-13 EM CCD camera with HCX PL APO (40X to 63X) objectives. Huygens Essential (Hilversum, Netherlands) deconvoluted algorithm was applied for images shown for Figures 2, 4, 5, 6 and 7. The ratio of staining intensity for NcoA4 to that for γ-tubulin at the centrosome in cells at different mitotic phases was determined by Volocity Quantification software version 5.3 (Perkin Elmer, Woodbridge, ON, Canada). For this study, the area occupied by the centrosomes was defined by γ-tubulin staining which was used as template to measure the corresponding NcoA4 staining intensity.

### Statistical analysis

The immunofluorescence images shown in this study are representative of 30 individual experiments. In each of these, all cells with mitotic figures exhibited NcoA4 staining localizing to the mitotic apparatus. In no case was an absence of NcoA4 staining at mitotic features observed. On average, 30-50 mitotic cells were examined per slide.

Data shown in Figure 9 are presented as mean ± SEM and were analyzed by Kruskal-Wallis One-Way ANOVA on Ranks followed by Dunn's multiple comparison method (SPSS 3.1 statistical software, Chicago, IL, USA) with alpha set at p<0.05.

## Results

### NcoA4 is evolutionarily conserved

**Figure 1:**
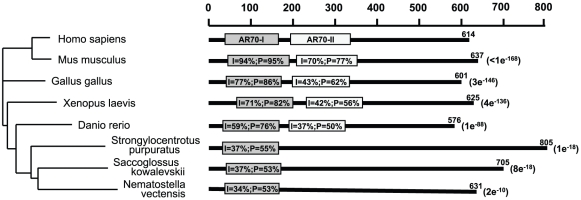
Evolutionary conservation of NcoA4 and the ARA70 domains. Blastp analysis indicates that vertebrate NcoA4 contains two ARA70 family domains designated as ARA70-I and ARA70-II. These domains have no significant sequence identity with one another. Relative to *H. sapiens,* ARA70-I sequence identity (I) ranges from 94% to 34% and positivity (P) from 95% to 53% and is present in all orthologs. In contrast, ARA70-II, which is restricted to vertebrates, ranges from 70% to 37% sequence identity and 77% to 50% positivity. The E-values indicated for each ortholog are based upon the full-length sequence and are relative to *H. sapiens*. A rooted phylogenetic dendrogram based upon full-length sequence similarity is shown on the left and is consistent with the accepted evolutionary history of metazoans.

*In silico* analysis of the human full-length amino acid sequence from NcoA4 indicated the presence of two ARA70 family domains located at amino acids 37–167 (ARA70-I) and 198–332 (ARA70-II). Blastp analysis of the full-length sequence or these two domains failed to identify related proteins within the human genome. However, orthologs of NcoA4 were identified in several metazoans ranging from cnidarians to mammals, with the exception of arthropods. The ARA70-I domain is conserved within all these species and is located within the predicted coiled-coil domain, whereas the ARA70-II domain is restricted to vertebrate species (Fig. 1). The conservation of the ARA70 domains within these orthologs suggest conservation of function(s).

### NcoA4 co-localizes with tubulin

**Figure 2:**
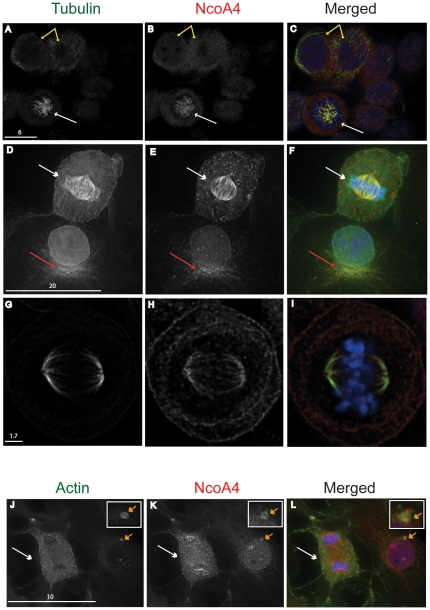
Co-localization of NcoA4 with tubulin and actin. T47D (**A** to **C** and **G** to **I**) or COS (**D** to **F** and **J** to **L**) cells were stained for NcoA4 and either α-tubulin (**A** to **C**), β-tubulin (**D** to **F**), acetylated tubulin (**G** to **I**) or actin (**J** to **L**). Cells were subjected to DAPI staining for visualization of chromatin (blue). Shown are representative images of non-mitotic cells (**A** to **C**, yellow arrow) and cells undergoing cell division (prophase: **A** to **C**, metaphase: **D** to **I**, and anaphase: **J** to **L**, white arrows). An aster formation is shown in **D** to **F** (red arrow). Yellow staining indicates overlapping NcoA4 and tubulin or actin (inset; orange arrow) localization. All images shown are of single optical slices obtained by confocal (**A** to **C** and **G** to **I**) or deconvolution (**D** to **F** and **J** to **L**) microscopy. [Bars are 1.7 µm-20 µm as indicated].

Coiled-coil domains which promote protein-protein interactions are common in proteins associated with the centrosome and microtubule network [14]. To determine if NcoA4 localizes to the cytoskeleton, dual label immunofluorescence deconvolution microscopy was performed on human breast cancer T47D and African green monkey kidney COS cells. Punctate yellow fluorescence representing overlapping NcoA4-α-tubulin localization was detected along the length of microtubules in non-mitotic cells (Fig. 2A to C). In addition, co-localization of NcoA4 withα-tubulin was detected in cells at prophase (Fig. 2A to C). Consistent with this finding, NcoA4 co-localized with β-tubulin (Fig. 2D to F) and acetylated tubulin (Fig. 2G to I), further confirming the association of NcoA4 with microtubules. In addition, during interphase, NcoA4 co-localized with the microtubule organizing centre (Fig. 2D to F). NcoA4 did not co-localize with microfilaments during mitosis (anaphase; Fig. 2J to L); however, co-localization of NcoA4 with actin was observed at the centrosome during interphase (inset; Fig. 2J to L).

### NcoA4 interacts with α-tubulin

**Figure 3:**
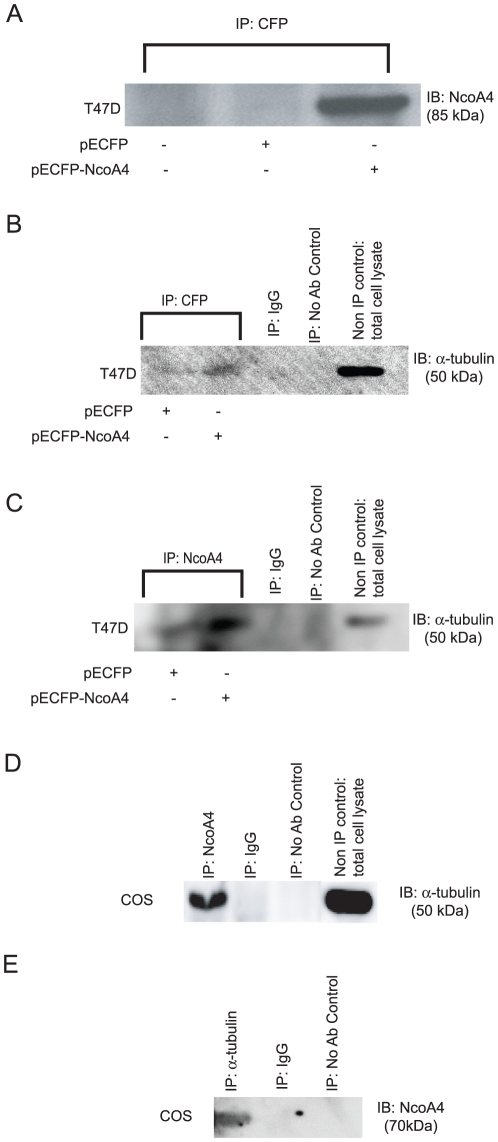
Interaction of NcoA4 with tubulin in T47D and COS cells. Interaction of NcoA4 with α-tubulin was demonstrated by co-immunoprecipitation. T47D (**A** to **C**) cells were transiently transfected with pECFP-NcoA4 or pECFP (empty vector) cDNA and cell lysates were collected 48 h later and incubated with anti-CFP (**A** and **B**), or anti-NcoA4 (**C**) antibody. The immunoblot was probed with NcoA4 (**A**), or α-tubulin (**B** and **C**) antibody. Cell lysates from non-transfected COS (**D** and **E**) cells were incubated with anti-NcoA4 (**D**) or anti-α-tubulin (**E**) and the resulting precipitates were subjected to 8% SDS-PAGE and transferred to an activated PVDF membrane. The immunoblot was probed with α-tubulin (**D**) or NcoA4 (**E**) antibody. COS and T47D total cell lysates (non-immunoprecipitated) were used as positive control (**B**, **C** and **D**).

To determine if α-tubulin interacts with NcoA4, lysates from T47D cells transfected with pECFP-NcoA4 expression vector were immunoprecipitated with CFP or NcoA4 antibody. The immunoprecipitates were resolved by SDS-PAGE and the resulting blots were probed with α-tubulin antibody. NcoA4overexpression in transfected cells was verified by the presence of an 85 kDa immunoreactive band corresponding to the predicted molecular weight of the NcoA4-CFP fusion protein (Fig. 3A). The interaction of α-tubulin with NcoA4 was verified by the presence of a 50 kDa immunoreactive band corresponding to α-tubulin in CFP- (Fig. 3B) or NcoA4- (Fig. 3C) immunoprecipitates. A 50 kDa immunoreactive band was observed in cells transfected with empty vector and immunoprecipitated with NcoA4 but not CFP antibody (Fig. 3C). This band likely represents tubulin interacting with endogenous NcoA4 expressed by these cells.

To verify endogenous NcoA4 interaction with α-tubulin, COS cell lysates were immunoprecipitated with NcoA4 or α-tubulin antibody and the resulting precipitates were subjected to Western blot analysis for α-tubulin or NcoA4 respectively. A 50 kDa immunoreactive band representing α-tubulin (Fig. 3D) and a 70 kDa immunoreactive band representing NχoA4 (Fig. 3E) were detected in NcoA4 and α-tubulin immunoprecipitates respectively, indicating that endogenous NcoA4 associates with α-tubulin.

### NcoA4 co-localizes with the mitotic apparatus

**Figure 4:**
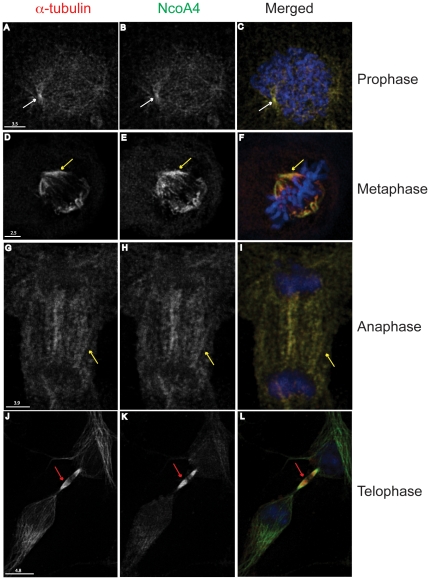
Co-localization of NcoA4 and α-tubulin at different stages of mitosis. COS cells were stained for NcoA4 (green) and α-tubulin (red) and examined by confocal microscopy. Cells were also subjected to DAPI staining for visualization of chromatin (blue). Shown are representative cells at different stages of cell division: **A** to **C**, prophase COS cell showing punctate NcoA4 staining associated with microtubules. White arrows indicate mitotic organization centers. **D** to **F**, metaphase T47D cell showing localization of NcoA4 to mitotic spindles (yellow arrow). **G** to **I**, anaphase COS cell showing localization of NcoA4 to mitotic spindles (yellow arrow). NcoA4 staining was diminished at the centrosomes at this stage. **J** to **L**, telophase COS cell showing strong NcoA4 staining at the midbodies (red arrow). Yellow indicates NcoA4-α-tubulin co-localization. The pattern of NcoA4 staining shown in these representative images were present in all cells undergoing mitosis. All images shown are of single optical slices. [Bars are 2.5 µm-4.8 µm as indicated].

NcoA4 co-localized with α-tubulin at all stages of cell division (Fig. 4). Dual labelling revealed that during prophase, NcoA4 co-localized with α-tubulin in spindle fibers extending from centrosomes and at the centrosomes (Fig. 4A to C). During metaphase (Fig. 4D to F) and anaphase (Fig. 4G to I), intense punctuate yellow fluorescence was associated with the aligned mitotic spindles. At late telophase, less intense yellow staining was detected along the microtubules with strong NcoA4 staining at the midbody (Fig. 4J to L).

**Figure 5:**
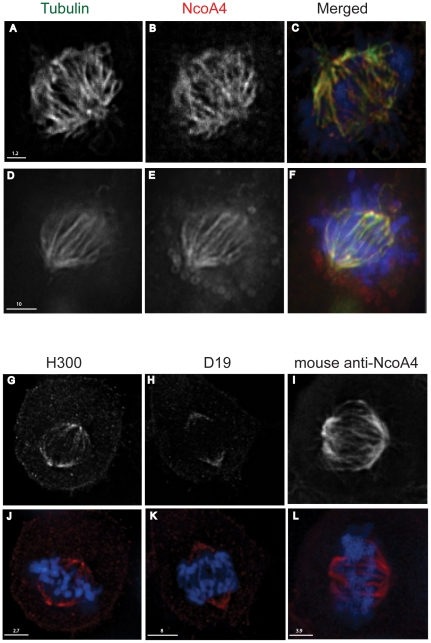
Co-localization of NcoA4 and α-tubulin in mammalian cell lines using various NcoA4 antibodies. COS (**A** to **C**) and ES2 (**D** to **F**) cells stained for NcoA4 (red) and α-tubulin (green) and examined by confocal and deconvolution microscopy, respectively. These images were generated using a rabbit polyclonal antibody raised against the first 300 amino acids (H300) of NcoA4. Panels **G** and **J**: COS cells stained for NcoA4 (red) using H300 antibody. Panels **H** and **K**: COS cells stained for NcoA4 (red) using a goat polyclonal antibody (D19). Panels **I** and **L**: T47D cells stained for NcoA4 using a mouse monoclonal antibody. Cells were also subjected to DAPI staining for visualization of chromatin (blue). Images shown in panels **G** to **L** were generated by confocal microscopy and are of representative cells undergoing metaphase. All images shown are of single optical slices. Yellow immunostaining indicates co-localization of NcoA4 with α-tubulin [Bars are 1.2 µm-10 µm as indicated].

**Figure 6:**
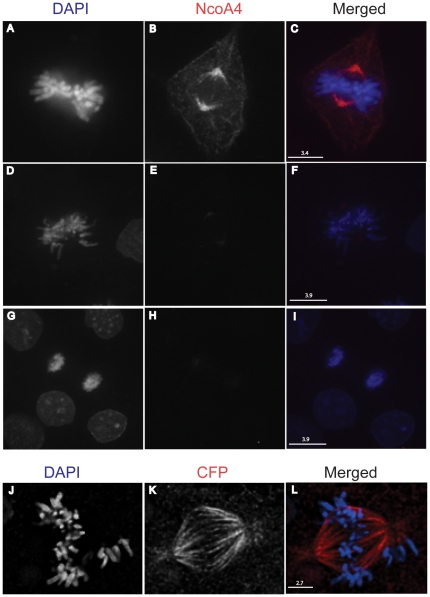
NcoA4 localization and preabsorption with corresponding immunizing peptide and localization of ECFP-tagged NcoA4 at the mitotic spindle. COS cells were stained for NcoA4 (red) using a goat polyclonal antibody (Q19) and examined by confocal microscopy. Cells were also subjected to DAPI staining for visualization of chromatin (blue). Shown is a representative image of a cell undergoing metaphase (**A** to **C**). Immunofluorescence with the antibody preabsorbed with the corresponding immunizing peptide (**D** to **I**). COS cells transiently transfected with pECFP-NcoA4 cDNA and stained for CFP-NcoA4 fusion protein using CFP antibody (**J** to **L**). All images shown are of single optical slices. [Bars are 2.7 µm to 3.9 µm as indicated].

The co-localization of NcoA4 with α-tubulin was observed with T47D human breast cancer cells (Fig. 2), and African green monkey kidney cells (COS; Figs. 2, 4 and 5A to C) and human ovarian cancer cells (ES2; Fig. 5D to F), indicating that the association of NcoA4 with microtubules during mitosis occurs in different mammalian cell types. The co-localization of NcoA4 with microtubules structures obtained using the rabbit polyclonal antibody raised against the first 300 amino acids of NcoA4 (Figs. 2, 4 and 5G and 5J) was replicated using three other commercially available antibodies raised against different regions of NcoA4 (a goat polyclonal antibody raised against amino acid residues 40 to 61; Fig. 5H and 5K, a mouse monoclonal antibody raised against amino acids 505 to 615; Fig. 5I and 5L, and a goat polyclonal raised against the internal region of NcoA4; Fig. 6A to C).

**Figure 7:**
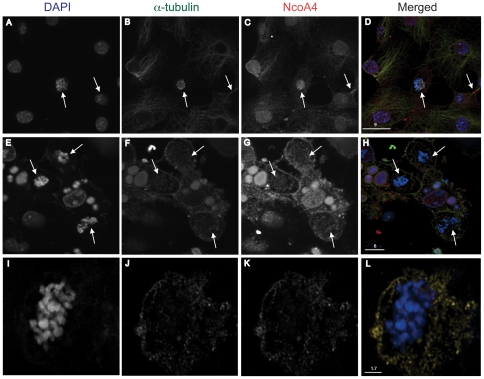
Co-localization of NcoA4 and α-tubulin in COS cells treated with nocodazole. COS cells untreated (**A** to **D**) or treated with 100 ng/ml nocodazole for 12 hours (**E** to **L**) were stained for NcoA4 (red) and α-tubulin (green) and examined by confocal microscopy. Cells were also subjected to DAPI staining for visualization of cell nuclei (blue). Yellow staining indicates NcoA4-α-tubulin co-localization. Images shown are of single optical slices. Arrows indicate mitotic figures. [Bars are 1.7 µm-9 µm as indicated].

The specificity of NcoA4 staining was verified by performing immunofluorescence using primary antibody preabsorbed with corresponding immunizing peptide which resulted in the loss of NcoA4 immunoreactivity (Fig. 6D to I). Localization of ECFP-tagged NcoA4 was also observed in the mitotic spindle in cells transiently transfected with pECFP-NcoA4 construct, further supporting the association of NcoA4 with microtubule structures (Fig. 6J to L). Depolymerization of microtubules with nocodazole resulted in dispersed NcoA4 staining, indicating that fully intact microtubule networks are not essential for co-localization of NcoA4 with α-tubulin (Fig. 7).

### Decreased centrosomal NcoA4 localization during mitotic progression

**Figure 8:**
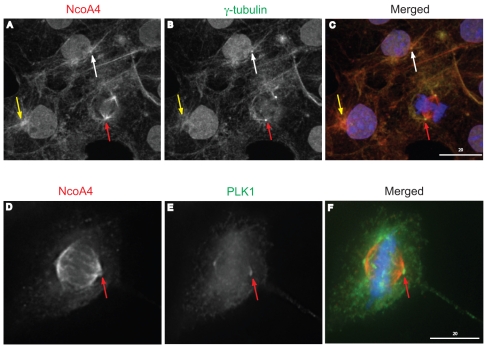
NcoA4 association with the centrosome during mitotic progression. Representative images of COS cells at different stages of mitosis stained for NcoA4 (red; **A,** and **D**), and γ−tubulin (green; **B**), or Plk1 (green; **E**). Merged images are shown in **C** and **F**. All images shown are of single optical slices obtained by confocal (**A** to **C**) or deconvolution (**D** to **F**) microscopy. Cells were also subjected to DAPI staining for visualization of chromatin (blue). NcoA4 was localized to aster formations (yellow arrows, **A** and **C**) and centrosomes (white arrows, **A** and **C**). Diminished NcoA4 staining at the centrosome during metaphase (red arrows, **A** and **D**) using γ-tubulin (**C**) or Plk1 (**F**) as a centrosomal marker. [Bars: 20 µm].

To address the localization of NcoA4 to centrosomes, COS cells were stained with NcoA4 and either γ-tubulin or Plk1. NcoA4 and γ-tubulin staining were detected at microtubule organizing centres and asters (Fig. 8A to C). Centrosomal co-localization of NcoA4 with γ-tubulin, a centrosomal marker, was evident during centrosomal segregation. However, the enrichment of NcoA4 at centrosomes was decreased at metaphase as evident by the diminished co-localization of NcoA4 with γ-tubulin (Fig 8A to C). This decreased co-localization was also demonstrated using Plk1 to demarcate the centrosome (Fig. 8D to F).

**Figure 9:**
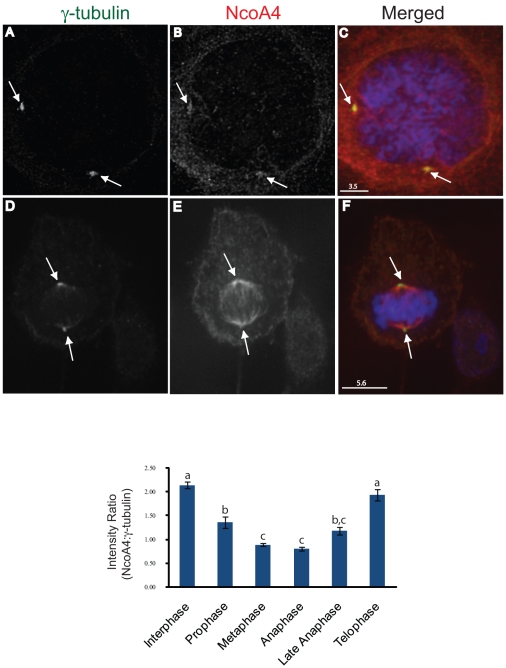
Dynamic changes of NcoA4 association with the centrosome during mitotic progression. COS cells were stained for NcoA4 (red; **B** and **E**), and γ-tubulin (green; **A** and **D**) and examined under confocal microscopy. Merged images are shown in **C** and **F** and all images shown are of single optical slices. Cells were also subjected to DAPI staining for visualization of chromatin (blue). The immunofluoresence intensity of centrosomal NcoA4 and γ-tubulin at different mitotic stages was determined by image analysis and the ratio summarized in the bar graph for cells at different stages of cell division. Data are presented as mean ± SEM of 27–147 centrosomes per group. Bars with different letters are statistically different from one another as determined by Kruskal Wallis ANOVA for Ranks followed by Dunn's multiple comparison method (p<0.05). [Bars are 3.5 µm and 5.6 µm as indicated]

The dynamic changes in centrosomal distribution of NcoA4 during mitosis was further examined by measuring the ratio of intensity of staining for NcoA4 to that of γ-tubulin at the centrosome in cells at different mitotic phases. Strong NcoA4 accumulation at the centrosomes was detected during interphase, and these levels decreased significantly in metaphase (Fig. 9). NcoA4 staining intensity at centrosomes during telophase was equivalent to interphase levels (Fig. 9).

## Discussion

Based upon *in silico* analysis of amino acid sequence similarities, NcoA4 is a unique protein with no paralogs. However, orthologs of this protein are found over a diverse range of metazoans, with significant sequence conservation within distinct regions referred to as ARA70-I and -II family domains. Although the functional significance of this evolutionary conservation is unknown, our findings demonstrate that NcoA4 is enriched at microtubule organizing centres, asters, mitotic spindles and midbodies, indicative of a potential role in spindle formation and function, and cytokinesis.

Other steroid receptor co-regulatory proteins have been shown to associate with microtubules. These include SRC3, and androgen receptor (AR)-associated proteins 67 (ARA67) and 24 (ARA24); however, of these only ARA24 has been shown to associate with the mitotic apparatus. Interaction of SRC3 with microtubules is thought to be necessary for its shuttling to the nucleus and for suppressing its intrinsic histone acetyltransferase activity within the cytoplasm [15]. ARA67, initially identified as amyloid precursor protein tail-1, due to its trafficking of amyloid, is a corepressor that retains AR in the cytoplasm through its binding to microtubules [16], [17]. ARA24 also has two independent actions that are determined in part by its subcellular localization. ARA24 functions within the nucleus as an AR coactivator [18], [19]. However, this Ran GTPase also localizes to the centrosome and mitotic spindle where it affects spindle function and microtubule-kinetochore interaction [20]–[25]. Since microtubules are critical for AR transport to the cell nucleus [26], it is possible that NcoA4 may facilitate microtubule transport of the AR as well as other nuclear receptors. However, similar to ARA24, our findings indicate that NcoA4 also localizes to the centrosome and mitotic spindle, suggesting it may have actions independent of its role as a nuclear receptor co-regulator.

Our results indicate a dynamic distribution of NcoA4 with components of the mitotic apparatus. Throughout cell division NcoA4 remains associated with the microtubule network whereas its association with centrosomes varies with the stage of mitosis. NcoA4 localizes to the mitotic organizing centre during interphase and early prophase but progressively declines within centrosomes during centrosomal segregation, reaching its lowest levels during metaphase and anaphase. NcoA4 staining intensity is increased in cells at late anaphase and telophase. Centrosomes are cellular organelles that evolved with metazoans and function to nucleate microtubules leading to spindle and aster formation. Therefore, it is intriguing that NcoA4 localizes to this organelle during spindle and aster maturation and declines when assembly of these structures is complete, raising the possibility that NcoA4 may contribute to microtubule nucleation.

In our phylogenetic analysis, orthologs for NcoA4 were not found in arthropods, raising the possibility that other proteins may compensate for the lack of this ortholog in this phylum. There are several examples where genes found in primitive organisms are lost in arthropods yet are present in mammals, a phenomenon often observed when genome comparisons are made which include highly derived organisms such as *Drosophila*. For example, fibrillar collagens are not encoded by insect genomes, but are present in cnidarians and advanced organisms such as mammals. Conversely, *Drosophila* express unique proteins such as CP309, a pericentrin-like coil-coiled domain-containing protein, which appears to be restricted to insects, and has been shown to nucleate microtubules in *Drosophila* [27].

The dynamic pattern of NcoA4 expression in the centrosomes during early mitosis, and its intense localization to the mitotic spindles progressing to the midbodies indicates that this protein may play an important role in cell division. The early centrosome and midbody localization of NcoA4 is similar to that reported for Cep55, a coiled-coil centrosomal protein that is required for cytokinesis [28]. Cep55 expression in the centrosome decreases following metaphase and reappears with the formation of the midbody, where it plays an essential role in cytokinesis. However, rather than localizing to the mitotic spindle during metaphase and anaphase, Cep55 diffuses throughout the cytoplasm. The localization of Cep55 is regulated by phosphorylation. Our *in silico* analysis of NcoA4 indicates several candidate phosphorylation sites, some of which are predicted to be targets of mitosis-associated kinases.

NcoA4 is expressed in oocytes and in tissues undergoing rapid proliferation during development, consistent with a role in spindle dynamics and mitosis [29], [30]. Altered expression of NcoA4 has also been reported in several cancers with expression of isoforms reported for both breast and prostate cancer [31]–[35]. Our work and that of others indicate that NcoA4 is expressed in all immortalized cell lines examined to date [4], [5], [32], [33]. While a role of NcoA4 in proliferation has been described in prostate cancer cell lines, these effects are dependent upon androgen and are likely due to NcoA4 functioning as an AR coregulatory protein [36], [37]. Our attempts to suppress NcoA4 expression with siRNA have thus far been unsuccessful, perhaps reflecting NcoA4 protein stability.

At present we cannot comment as to whether the localization of NcoA4 to microtubules and the mitotic apparatus results from a direct or indirect interaction with tubulin. Immunoprecipitation of cell lysates with antibodies to either NcoA4 or tubulin co-precipitate multiple proteins (data not shown), raising the possibility that NcoA4 may associate with these structures through intermediately proteins.

In the present study, we provide evidence that suggests NcoA4 may have functions in addition to its role as nuclear receptor coactivator. The subcellular distribution we report suggests that NcoA4 may regulate multiple aspects of microtubule activity during mitosis. NcoA4 localization to midbodies suggests that NcoA4 may influence events associated with cytokinesis. Further studies are required to determine the precise functions of NcoA4 during cell division and whether it plays a role in chromosomal segregation and cytokinesis during normal and pathological development.
